# Changes in Choroidal Structure Associated with Idiopathic Macular Hole Border Morphology

**DOI:** 10.3390/jcm14186362

**Published:** 2025-09-09

**Authors:** Hiroaki Endo, Hiroto Terasaki, Shozo Sonoda, Yuki Ito, Satoshi Katsuta, Satoru Kase

**Affiliations:** 1 Department of Ophthalmology, Teine Keijinkai Hospital, Sapporo 006-0811, Japan; bananapony@gmail.com (H.E.);; 2 Department of Ophthalmology, Kagoshima University, Kagoshima 890-0065, Japansonosho0110@gmail.com (S.S.); 3 Department of Ophthalmology, Nara Medical University, Kashihara 634-8521, Japan

**Keywords:** idiopathic macular hole, optical coherence tomography, choroidal structure, choriocapillaris, L/C ratio

## Abstract

**Objectives**: To investigate the association between the hole border morphology and choroidal structure in idiopathic macular hole (IMH) patients and its impact on visual outcomes. **Methods**: A retrospective case–control study of 34 IMH eyes and 34 control eyes was conducted. Spectral-domain optical coherence tomography (SD-OCT) was used to categorize the patients into groups with smooth or bumpy hole borders. Based on this classification, a further evaluation was conducted of MH morphology and choroidal structure, both before a vitrectomy and at 1 and 2 months post-surgery. The choriocapillaris, Sattler’s layer, and Haller’s layer were the divisions of each choroidal vascular layer. Then, binarization techniques were employed to calculate the choroidal area (CA), luminal area (LA), stromal area (SA), and central choroidal thickness (CCT). The L/C ratio was established as the ratio of LA to CA. **Results**: In the patients with IMH, the choroidal structure was associated with the morphology of the hole border. In particular, the eyes with bumpy hole borders were significantly correlated with reduced choroidal thickness and total choroidal area, as well as a reduced L/C ratio in the choriocapillaris. After surgery, visual acuity improved in both groups, but the patients with smooth hole borders achieved earlier and higher levels of visual recovery. The multivariate analysis suggested that a bumpy hole border and the basal hole diameter were independent predictors of postoperative choriocapillaris recovery. **Conclusions**: Bumpy idiopathic macular hole borders are associated with impaired choroidal vasculatures, particularly in the choriocapillaris, which may be a contributing factor to delayed visual acuity recovery post-surgery.

## 1. Introduction

An idiopathic macular hole (IMH), the most common type of macular hole (MH), is characterized by a loss of neuroretinal tissue in the fovea [1,2]. Although vitreoretinal interface abnormalities and traction are established contributors to MH development, the underlying pathophysiological mechanisms remain incompletely understood.

Emerging evidence suggests that choroidal alterations, both anatomical and physiological, may play a role in IMH progression. Aras et al. reported significantly reduced blood flow and velocity in the foveal choriocapillaris of IMH eyes compared with healthy controls [3]. Similarly, Zhang et al. found notable choroidal thinning in IMH eyes compared to their contralateral eyes, and proposed that morphological changes in the choroid may be involved in MH formation [4]. Our previous work using choroidal optical coherence tomography (OCT) with novel binarization software demonstrated structural alterations in the choriocapillaris of IMH patients. Furthermore, our findings indicated that postoperative choroidal recovery may contribute to visual improvement [5]. Together, these data suggest that, beyond the traction theory proposed by Gass et al. [1,2], morphophysiological changes in the choroid may play a pivotal role in both IMH development and visual recovery.

Recent studies have also indicated that the morphology of the MH border—classified as “smooth” or “bumpy”—affects postoperative outcomes [6]. A “smooth” border is generally associated with better visual recovery, while a “bumpy” border often indicates severe photoreceptor damage and a worse prognosis. However, the relationship between the MH border morphology and underlying choroidal structure remains unclear. Therefore, this study aims to compare the choroidal features of IMHs with smooth and bumpy borders to clarify their pathophysiological differences and implications for visual prognosis. We hypothesize that this novel classification based on border morphology is associated with the choroidal vascular changes suggested by prior research. This approach is expected to offer a new framework for clinical decision-making by linking an OCT finding to the underlying disease mechanisms.

## 2. Materials and Methods

### 2.1. Ethics Statement

A retrospective review of patient cases was conducted at Teine Keijinkai Hospital. The study design was formally approved by the Teine Keijinkai Hospital’s Institutional Review Board (IRB approval number: 2-024423-00), and all procedures were performed in accordance with the ethical standards of the Declaration of Helsinki. Due to the retrospective nature of the study, written informed consent was waived. Instead, an opt-out approach was adopted, and the details of the study were disclosed on the hospital’s website. Patients were given the opportunity to decline participation.

### 2.2. Study Participants

This study included patients with an IMH who visited the Retinal Vitreous Center at Teine Keijinkai Hospital between June 2019 and August 2024. All the participants underwent comprehensive ophthalmic evaluations, including assessments of visual acuity, intraocular pressure, slit-lamp biomicroscopy, and a fundus examination. The inclusion criteria were as follows: (1) age ≥ 18 years; (2) presence of an IMH classified as small–medium (<400 μm) or large (>400 μm); (3) underwent a phacovitrectomy; and (4) completed a 2-month postoperative follow-up. The exclusion criteria included the following: (1) diabetes mellitus or uncontrolled hypertension; (2) a secondary or traumatic macular hole; (3) high myopia (refractive error > −6 D or axial length > 26 mm); (4) media opacity affecting the OCT image quality; and (5) a history of glaucoma, trauma, uveitis, other retinal diseases, or prior ocular surgery. The data were collected from electronic medical records and included visual symptoms and their duration; the best-corrected visual acuity (BCVA) in logarithm of the Minimum Angle of Resolution (logMAR), before surgery and at 1 and 2 months postoperatively; and the surgical details (pars plana vitrectomy alone or combined with cataract extraction). The macular hole staging was based on clinical and intraoperative findings. The duration of illness was defined as the period from the onset of subjective symptoms, not the period from diagnosis to surgery. Control subjects were selected from the hospital’s OCT database and matched for age, sex, refractive error, and axial length.

### 2.3. OCT Image Acquisition

Spectral-domain OCT devices (Cirrus HD-OCT 5000, Carl Zeiss Meditec, Dublin, CA, USA) were used to acquire the OCT images. The macular imaging was conducted using a horizontal raster scan, with a 9 mm scan line centered at the foveal region. To minimize diurnal variations, the OCTs were conducted between 9:00 a.m. and 11:00 a.m. Experienced technicians acquired the images, which were then evaluated for a signal strength of 7/10 or higher. Additionally, for the analysis utilizing Kago-Eye2 software, the OCT images were exported as TIFF files [7]. Two blinded researchers, H.E. and Y.I., independently evaluated each measurement.

### 2.4. Retinal Image Analysis Using OCT Images

**Figure 1:**
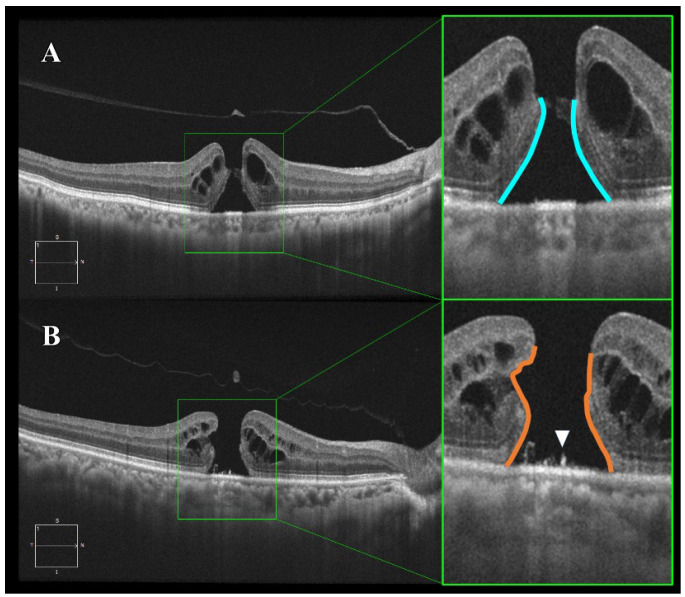
Optical coherence tomography (OCT) images of smooth and bumpy idiopathic macular hole (IMH) borders. Horizontal B-scan images obtained by spectral-domain OCT. (**A**) Smooth border: The IMH exhibits a uniform and regular border with relatively mild photoreceptor damage (blue line). (**B**) Bumpy border: The IMH shows a non-uniform and irregular border with severe photoreceptor damage due to disruption of the external limiting membrane and ellipsoid zone (orange line). Additionally, supra-retinal pigment epithelium (RPE) granular deposits are observed (indicated by the white triangle).

Full-thickness MHs were classified based on the International Vitreomacular Traction Study (IVTS) Group criteria [8]. The edge morphology was divided into two categories—“smooth” and “bumpy”—as described by Govetto et al. [6]. “Smooth” referred to a hole with regular, even borders, whereas “bumpy” described an irregular, uneven contour (Figure 1).

**Figure 2:**
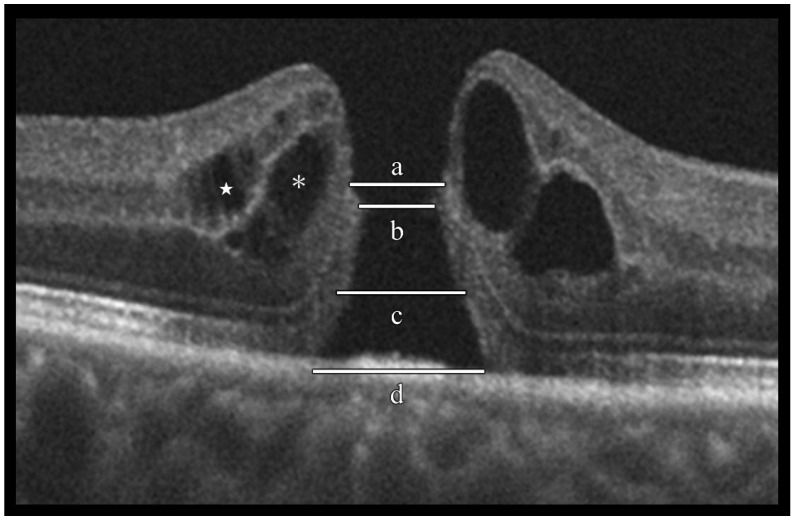
Evaluation of idiopathic macular hole (IMH) morphological characteristics using optical coherence tomography (OCT). Spectral-domain OCT horizontal B-scan images were used to evaluate the following morphological characteristics of IMHs: (a) external limiting membrane (ELM) defect length, (b) minimum hole diameter, (c) ellipsoid zone (EZ) defect length, (d) basal hole diameter, inner-layer cyst (located in the inner nuclear layer, indicated by the white star), and outer-layer cyst (located in the outer plexiform layer to the Henle fiber layer, indicated by an asterisk).

The OCT images obtained before and after surgery were used to evaluate the following parameters: the minimum and basal hole diameters, the inner and outer macular fluid, the ELM and EZ defect lengths, and the presence of supra-RPE granular deposits (Figure 2). The minimum hole diameter was defined as the shortest distance between the internal edges of the MH at the level of the inner retina, while the basal hole diameter was measured at the level of the retinal pigment epithelium (RPE). The inner macular fluid was defined as intraretinal cystoid cavities within the inner nuclear layer (INL), and the outer macular fluid as those located in the Henle fiber layer–outer nuclear layer (HFL–ONL). The lengths of ELM and EZ defects were determined by the horizontal extent of signal disruption in the corresponding hyperreflective layers. Supra-RPE granular deposits were identified as multiple rounded hyperreflective foci located on the RPE at the base of the MH.

### 2.5. Choroidal Image Analysis Using OCT Images

**Figure 3:**
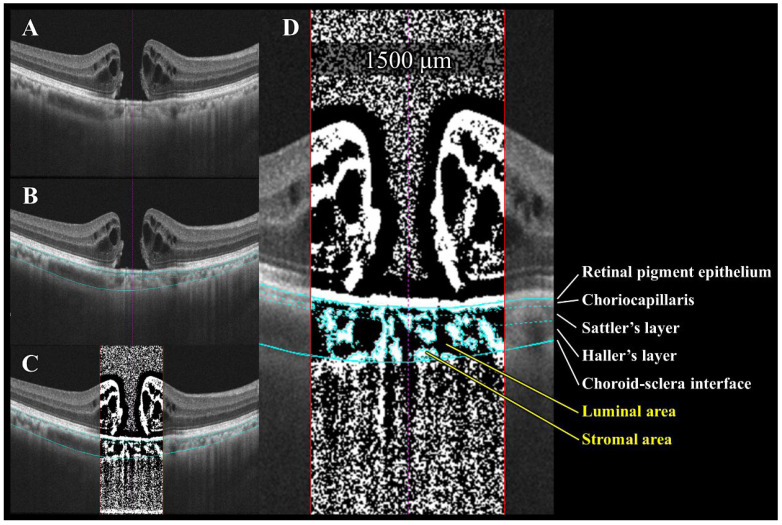
Semi-automated choroidal binarization of idiopathic macular hole (IMH) with bumpy border. (**A**) Enhanced depth imaging optical coherence tomography (EDI-OCT) image of IMH before surgery. (**B**) Light blue lines delineate following interfaces from top to bottom: retinal pigment epithelium (RPE)–choriocapillaris, choriocapillaris–Sattler layer, Sattler layer–Haller layer, and choroid–sclera. (**C**) EDI-OCT image binarized using Niblack’s method. Dark pixels represent luminal area, while light pixels represent stromal area. (**D**) Quantification of luminal and stromal areas within each 1500 μm wide choroidal vascular layer and calculation of luminal/choroidal area ratio (L/C ratio).

The choroidal structure was assessed using established binarization methods [7,9] (Figure 3). As detailed previously [7,9], the EDI-OCT images were imported into Kago-Eye2 software, and a 1500 μm wide region centered on the fovea was delineated. The choroidal boundaries were manually identified, followed by a semi-automated segmentation of the choriocapillaris, and Sattler’s and Haller’s layers. The binarized images yielded measurements of choroidal area (CA), luminal area (LA), stromal area (SA), and central choroidal thickness (CCT). Bright pixels corresponded to the SA and dark pixels to the LA. The L/C ratio was calculated as the LA divided by the CA. The choice to use a 1500 μm wide region for choroidal analysis in this study was based on two primary reasons. First, the main pathological changes in the idiopathic macular holes (IMHs) were concentrated within this range, which allowed us to accurately evaluate the changes highly relevant to the lesion. Second, this analytical range ensured consistency with prior research. By adopting the same range used in many published studies, our data can be directly compared with past findings, demonstrating the continuity of our long-term research outcomes. All the measurements in this study were performed independently by two researchers (H.E. and Y.I.). To assess the inter-grader agreement, we used the intraclass correlation coefficient (ICC) and Bland–Altman analysis. After confirming that there was no significant difference between the two sets of measurements (verified by the absence of fixed and proportional biases on Bland–Altman plots), the average of the two measurements was adopted as the final data for analysis. This method ensured the objectivity and reliability of our measurements.

### 2.6. Surgical Procedure

All the patients received a standard 25-gauge 3-port pars plana vitrectomy (PPV) with concurrent cataract surgery, using the Constellation Vision System (Alcon Laboratories Inc., Fort Worth, TX, USA). A core vitrectomy and, if needed, artificial posterior vitreous detachments were performed. Brilliant Blue G was used to stain and peel the internal limiting membrane (ILM). A fluid–air exchange was followed by an SF_6_ injection into the vitreous cavity. The patients were instructed to maintain a prone position until the OCT confirmed MH closure.

### 2.7. Statistical Analysis

The statistical analysis was conducted using SPSS version 30 (IBM Corporation, Armonk, NY, USA). The data were expressed as the mean ± standard deviation. The differences in various characteristics between the IMH patients and controls were assessed using the Mann–Whitney U test. A chi-square test or Fisher’s exact test was used to compare the categorical variables. Comparison of choroidal structures between the control group and the IMH group before surgery was analyzed using the Kruskal–Wallis test and the Steel–Dwass test. The changes in preoperative and postoperative visual acuity and chorioretinal structure were evaluated using the Friedman test. When a significant difference was found with the Friedman test, a post hoc analysis was performed using the Wilcoxon signed-rank test for pairwise comparisons, and the *p* values were adjusted using the Bonferroni correction to account for multiple comparisons. *p* values less than 0.05 were considered statistically significant.

## 3. Results

### 3.1. Demographics

A total of 89 eyes were initially enrolled in this IRB-approved study. Fifty-five eyes were excluded due to a short follow-up (<2 months, 40 eyes), missing OCT data (7 eyes), or presence of diabetes mellitus (8 eyes), leaving 34 IMH eyes from 33 patients eligible for analysis. An equal number of healthy control eyes (n = 34) were also included. Table 1 summarizes the demographic and baseline data. No significant differences were observed between the groups in terms of age, sex, refractive error, axial length, hypertension prevalence, systolic blood pressure (SBP), or diastolic blood pressure (DBP). However, the intraocular pressure was significantly higher in the IMH group.

The preoperative MH staging showed 26.5% stage 2, 47.1% stage 3, and 26.5% stage 4. The IMH eyes had significantly worse baseline BCVA. The preoperative morphologic values were as follows: minimum hole diameter, 356 ± 137 μm; basal hole diameter, 767 ± 287 μm; ELM defect length, 496 ± 214 μm; and EZ defect length, 588 ± 276 μm. The prevalence of inner macular fluid, outer macular fluid, and supra-RPE granular deposits was 55.9%, 97.1%, and 50.0%, respectively. In comparison with the smooth group, the bumpy-border eyes exhibited more severe pathology. They showed a significantly longer symptom duration (*p* = 0.004) and a more advanced MH stage (*p* = 0.03). Furthermore, these eyes presented with larger hole diameters (*p* = 0.007 and *p* = 0.009) and more frequent inner macular fluid (*p* < 0.001), along with longer ELM/EZ defects (*p* = 0.004 and *p* = 0.001) and more frequent supra-RPE granular deposits (*p* = 0.01).

### 3.2. Reproducibility Assessment of Choroidal Structural Parameters

**Figure 4:**
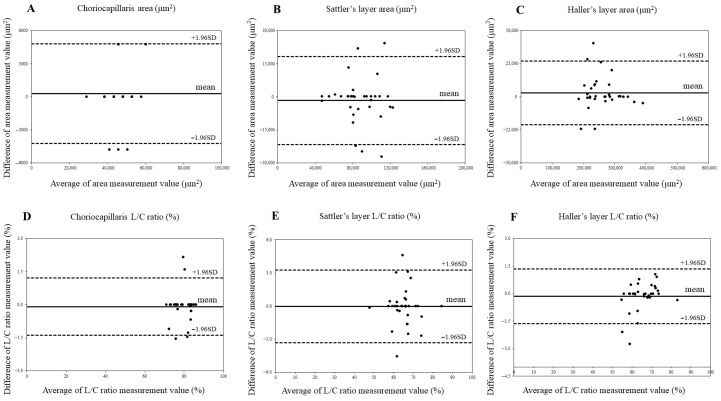
Agreement between choroidal area and luminal/choroidal area ratio (L/C ratio) in control eyes using Bland–Altman plots. (**A**) Choriocapillaris area in control eyes. (**B**) Sattler’s layer area in control eyes. (**C**) Haller’s layer area in control eyes. (**D**). Choriocapillaris L/C ratio in control eyes. (**E**) Sattler’s layer L/C ratio in control eyes. (**F**) Haller’s layer L/C ratio in control eyes.

**Figure 5:**
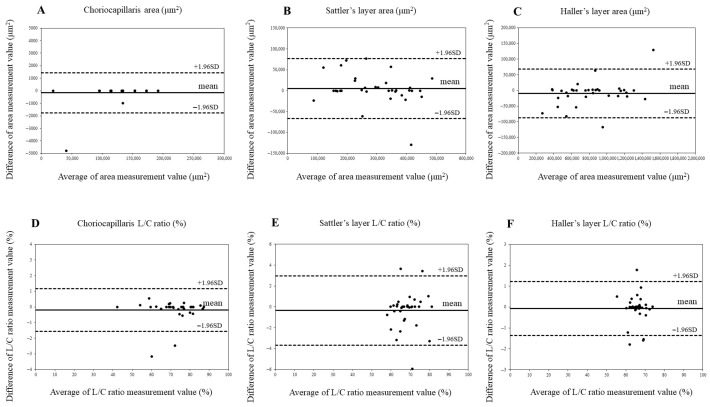
Agreement between choroidal area and luminal/choroidal area ratio (L/C ratio) in idiopathic macular hole (IMH) eyes using Bland–Altman plot. (**A**) Choriocapillaris area in IMH eyes. (**B**) Sattler’s layer area in IMH eyes. (**C**) Haller’s layer area in IMH eyes. (**D**) Choriocapillaris L/C ratio in IMH eyes. (**E**) Sattler’s layer L/C ratio in IMH eyes. (**F**) Haller’s layer L/C ratio in IMH eyes.

The choroidal measurement reproducibility was assessed using an ICC and Bland–Altman analysis (Table 2, Figure 4 and Figure 5). ICC values > 0.9 indicated high reliability. No significant fixed bias was detected for the choroidal area or L/C ratio in either group. Likewise, proportional bias was not observed. These findings confirm consistent measurements by the evaluators.

### 3.3. Preoperative Choroidal Structure Analysis

The binarization analysis of preoperative OCT images (Table 3) showed that the IMH eyes had significantly lower choriocapillaris CA, LA, and L/C ratios, and a higher SA compared with the controls. In the bumpy-border eyes, the CA, LA, and L/C ratios in the full choroid were significantly reduced compared to both the controls and the smooth-border eyes. Additionally, the LA and L/C ratios in the bumpy eyes were significantly lower than in the smooth eyes. In Haller’s layer, the CA and LA were lower in the bumpy eyes than in the controls, and the CCT was significantly thinner in the IMH eyes overall.

### 3.4. Postoperative Changes in BCVA and Retinal Structure

**Figure 6:**
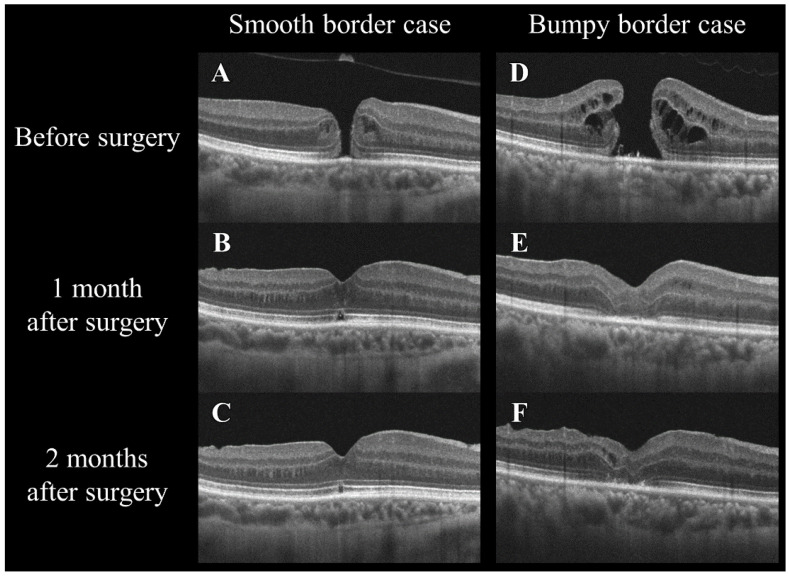
Changes in the optical coherence tomography morphology of smooth and bumpy borders in idiopathic macular holes before and after a vitrectomy. The left column (**A**–**C**) shows a smooth border case, and the right column (**D**–**F**) shows a bumpy border case. The images in the top row (**A**,**D**) were taken before surgery, those in the middle row (**B**,**E**) at 1 month after surgery, and those in the bottom row (**C**,**F**) at 2 months after surgery. Note the changes in the macular hole configuration and the adjacent retinal layers over time in both types of cases.

All the MHs were closed postoperatively. As shown in Table 4 and Figure 6, both groups experienced significant BCVA improvement at 2 months. The smooth-border group demonstrated earlier and more sustained gains in visual acuity. This trend was also observed in the ELM and EZ defect lengths, with significant early improvement in the smooth group.

### 3.5. Changes in Choroidal Structure in IMH Eyes After Surgery

Table 5 presents the postoperative choroidal changes in the IMH eyes. The total choroidal area remained significantly larger in the smooth-border eyes throughout the follow-up. Both groups showed an increase in the L/C ratio postoperatively. At 2 months, the L/C ratio rose by 15.8% in the smooth eyes and by 23.5% in the bumpy eyes, though the smooth group maintained a higher absolute value over time. The choriocapillaris thickness and L/C ratio increased after surgery in both groups (Table 6). No significant postoperative changes were detected in Sattler’s or Haller’s layers (Table 7 and Table 8). The CCT was significantly thinner in the bumpy-border eyes at 2 months, with no consistent postoperative changes observed in the smooth group.

### 3.6. Predictors of Postoperative Prognosis

A multiple regression analysis (Table 9) identified a bumpy hole border morphology and larger basal hole diameter as significant predictors of postoperative improvement in the L/C ratio of the choriocapillaris.

These results show that the bumpy border group exhibited a larger hole diameter and features of chronicity, as well as poorer postoperative choroidal recovery. A summary of these key findings is presented in Table 10.

## 4. Discussion

Our study reveals a close association between the morphology of the hole border in IMH and the choroidal vascular structure, particularly the perfusion status of the choriocapillaris. In the eyes with a bumpy border, there was a significant decrease in the choroidal thickness and total choroidal area, along with a significant reduction in the L/C ratio of the choriocapillaris, compared to the eyes with a smooth border. These findings suggest that a bumpy border morphology is associated with more severe choroidal blood flow impairment, emphasizing the crucial role of the choroid in the pathophysiology of IMH.

The difference in the postoperative visual function outcomes between the two groups can be explained by these choroidal structural differences. Our results suggest that while surgery may improve choroidal blood flow, the extent of preoperative photoreceptor damage is a key determinant of final visual acuity. In other words, even if the blood flow improves after surgery, the recovery of severely damaged photoreceptors is difficult, resulting in a limited improvement in visual function. Recent OCTA studies have shown that choriocapillaris blood flow increases after IMH surgery, which correlates with improved visual acuity [10,11,12]. However, in our bumpy border group, visual recovery was limited despite improved choriocapillaris perfusion after surgery. This is likely because the bumpy border is associated with severe and irreversible photoreceptor damage, as indicated by past histological studies [6,13]. This disparity between blood flow improvement and functional recovery highlights the importance of evaluating the preoperative state of photoreceptors for predicting the prognosis of IMH.

Our results suggest that a bumpy border morphological classification is not just a difference in shape, but a comprehensive indicator reflecting the chronicity and severity of the disease. Our results, similar to reports from previous studies [6,14,15,16], show that the bumpy border group had a longer disease duration, a larger hole diameter, and many features suggestive of chronicity, such as ELM/EZ defects, intraretinal fluid, and supra-RPE granular deposits. The results from the multiple regression analysis show that a bumpy border classification and the basal hole diameter were independent and significant predictors of postoperative choroidal blood flow recovery. Supra-RPE granular deposits and the MH stage were not independent predictors, likely because the predictive value of supra-RPE granular deposits is aggregated within the broader “bumpy border” classification. Therefore, our results emphasize the importance of evaluating the preoperative state of photoreceptors, in addition to the postoperative blood flow improvement, for predicting the prognosis of IMH. We believe that this is a significant contribution of our study that cannot be fully captured by studies using OCTA alone.

### 4.1. Clinical Implications

As our study demonstrates, the preoperative OCT findings, particularly the morphology of the hole border, serve as a simple yet crucial predictor for postoperative choroidal recovery. Since IMHs with a “bumpy” border tend to show poorer choroidal recovery compared to those with a “smooth” border, this information is valuable for prognostic counseling and managing patient expectations. This finding provides a new framework for stratifying IMH based on the border morphology and when considering individualized treatment strategies. For example, a more cautious postoperative management approach may be warranted for IMHs with a bumpy border to promote better long-term recovery.

### 4.2. Limitations

This study identified alterations in the choroidal vascular structures of eyes with IMH; however, several limitations must be considered. First, the sample size was relatively modest (34 IMH eyes and 34 control eyes), which is a limitation particularly for the subgroup analyses between the “smooth” and “bumpy” borders. This may limited the statistical power and generalizability of our findings, and further studies with larger populations are warranted to validate the results. Second, the follow-up period in this study was limited to two months. Given that visual and structural recovery of the choroid in IMH is known to continue for 6 to 12 months or longer, our conclusions regarding prognosis and structural recovery should be considered preliminary. Future studies with longer follow-up periods are essential to evaluate the long-term prognosis and more complete structural recovery. Third, EDI-OCT imaging has intrinsic limitations, as deeper signal penetration may be affected by the properties of overlying tissues. Comparative studies using both EDI-OCT and SS-OCT could help address these biases. Lastly, it remains uncertain whether the observed choriocapillaris changes are specific to IMH repair. Future research should investigate choroidal alterations in vitrectomized eyes with other underlying conditions.

## 5. Conclusions

Evaluating the morphology of the hole border in an IMH eye can be a crucial clinical tool for predicting both the postoperative choroidal circulation recovery and visual function outcomes. A bumpy border is a significant sign that suggests severe choroidal blood flow impairment and irreversible photoreceptor damage. Therefore, clinicians should consider that patients with this finding may have limited visual function recovery, even after achieving anatomical success.

## Figures and Tables

**Table 1:** Comparison of clinical characteristics between control eyes and idiopathic macular holes (IMHs) with different border morphologies.

	Control Eyes	IMH Eyes	*p* Value *	IMH with Smooth Borders	IMH with Bumpy Borders	*p* Value †
Number of eyes	34	34		23	11	
Age, years	67.0 ± 8.0	66.3 ± 7.9	0.68 ‡	65.7 ± 8.5	67.7 ± 6.2	0.45 ‡
Gender, male/female	10/24	10/24	0.99 **	7/16	3/8	0.59 ††
BCVA-logMAR	−0.04 ± 0.08	0.76 ± 0.27	**<0.001** ‡	0.71 ± 0.28	0.86 ± 0.23	0.17 ‡
IOP, mmHg	13.9 ± 1.9	15.0 ± 2.1	**0.03** ‡	15.1 ± 2.1	14.7 ± 2.0	0.97 ‡
SE, diopters	−0.55 ± 1.57	−0.53 ± 2.09	0.59 ‡	−0.52 ± 2.06	−0.56 ± 2.15	0.78 ‡
AL, mm	23.75 ± 0.88	23.85 ± 0.84	0.77 ‡	23.72 ± 0.79	24.12 ± 0.89	0.25 ‡
HT, %	29.4	35.3	0.60 **	34.8	36.4	0.61 ††
SBP, mmHg	123 ± 13	122 ± 13	0.24 ‡	122 ± 14	122 ± 10	0.65 ‡
DBP, mmHg	70 ± 10	73 ± 10	0.29 ‡	74 ± 10	72 ± 9	0.63 ‡
Retinal characteristics						
Duration of symptoms, days	-	50 ± 56		28 ± 26	96 ± 73	**0.004** ‡
MH stage 2:3:4, eyes	-	9:16:9		8:12:3	1:4:6	**0.03** **
Minimum hole diameter, μm	-	356 ± 137		301 ± 90	471 ± 146	**0.007** ‡
Basal hole diameter, μm	-	767 ± 287		666 ± 216	978 ± 303	**0.009** ‡
Inner macular fluid, %	-	55.9		34.8	100.0	**<0.001** ††
Outer macular fluid, %	-	97.1		95.7	100.0	0.68 ††
ELM defect length, μm	-	496 ± 214		430 ± 198	633 ± 179	**0.005** ‡
EZ defect length, μm	-	588 ± 276		471 ± 162	844 ± 286	**0.001** ‡
Supra-RPE granules, %	-	50.0		34.8	81.8	**0.01** **

BCVA, best-corrected visual acuity; IOP, intraocular pressure; SE, spherical equivalent; AL, axial length; HT, hypertension; SBP, systolic blood pressure; DBP, diastolic blood pressure; ELM, external limiting membrane; EZ, ellipsoid zone; RPE, retinal pigment epithelium. * *p* value for comparison between control eyes and IMH eyes. † *p* value for comparison between IMHs with smooth borders and IMHs with bumpy borders. ‡ Mann–Whitney U test. ** Chi-square test. †† Fisher’s exact test. Statistically significant values are highlighted in bold.

**Table 2:** Inter-examiner reliability of choroidal segmentation in idiopathic macular holes (IMHs) and control eyes.

			Relative Reliability (ICC)				Bland–Altman Analysis (Fixed Bias)		Bland–Altman Analysis (Proportional Bias)	
			ICC (Single)	*p* Value	ICC (Mean)	*p* Value	CI 95%	*p* Value	*R*	*p* Value
Control eyes	Choriocapillaris (CC)	CC area	0.9280	**<0.001**	0.9627	**<0.001**	−535~1099	0.49	0.09	0.63
		L/C ratio	0.9881	**<0.001**	0.9970	**<0.001**	−0.14~0.33	0.44	−0.03	0.88
	Sattler’s layer (SL)	SL area	0.8705	**<0.001**	0.9308	**<0.001**	−1889~5351	0.34	−0.17	0.35
		L/C ratio	0.9617	**<0.001**	0.9805	**<0.001**	−0.56~0.65	0.90	−0.08	0.65
	Haller’s layer (HL)	HL area	0.9689	**<0.001**	0.9684	**<0.001**	−1470~7191	0.19	−0.02	0.94
		L/C ratio	0.9927	**<0.001**	0.9963	**<0.001**	−0.13~0.42	0.29	0.41	0.17
IMH eyes	Choriocapillaris (CC)	CC area	0.9997	**<0.001**	0.9999	**<0.001**	−121~461	0.24	0.25	0.12
		L/C ratio	0.9974	**<0.001**	0.9987	**<0.001**	−0.05~0.44	0.11	−0.16	0.36
	Sattler’s layer (SL)	SL area	0.9475	**<0.001**	0.9731	**<0.001**	−8090~17835	0.45	−0.21	0.23
		L/C ratio	0.9589	**<0.001**	0.9580	**<0.001**	−0.26~0.94	0.25	0.17	0.33
	Haller’s layer (HL)	HL area	0.9922	**<0.001**	0.9961	**<0.001**	−4604~23415	0.18	0.17	0.35
		L/C ratio	0.9844	**<0.001**	0.9921	**<0.001**	−0.16~0.31	0.53	−0.11	0.53

ICC, intraclass coefficient; CI 95%, 95% confidence interval; L/C ratio, luminal/choroidal ratio. Statistically significant values are highlighted in bold.

**Table 3:** Comparison of choroidal characteristics in idiopathic macular holes with smooth versus bumpy borders (IMHs).

	Control Eyes	IMH with Smooth Borders	IMH with Bumpy Borders	Kruskal–Wallis Test (*p* Value)	Steel–Dwass Test (*p* Value): Control–Smooth	Steel–Dwass Test (*p* Value): Control–Bumpy	Steel–Dwass Test (*p* Value): Smooth–Bumpy
**Total choroid**							
CA, ×10^3^ μm^2^	393 ± 51	396 ± 117	301 ± 60	**<0.001**	0.79	**<0.001**	**0.04**
LA, ×10^3^ μm^2^	263 ± 40	260 ± 77	190 ± 38	**<0.001**	0.94	**<0.001**	**0.02**
SA, ×10^3^ μm^2^	129 ± 19	137 ± 41	112 ± 22	0.07	NA	NA	NA
L/C ratio, %	66.9 ± 3.7	65.3 ± 3.0	63.0 ± 1.4	**0.002**	0.32	**0.002**	**0.01**
**Choriocapillaris**							
CA, ×10^3^ μm^2^	46 ± 6	37 ± 6	32 ± 6	**<0.001**	**<0.001**	**<0.001**	0.07
LA, ×10^3^ μm^2^	37 ± 5	23 ± 6	15 ± 5	**<0.001**	**<0.001**	**<0.001**	**0.008**
SA, ×10^3^ μm^2^	9 ± 2	15 ± 3	17 ± 3	**<0.001**	**<0.001**	**<0.001**	0.19
L/C ratio, %	79.6 ± 4.3	60.1 ± 9.2	46.9 ± 9.2	**<0.001**	**<0.001**	**<0.001**	**0.005**
**Sattler’s layer**							
CA, ×10^3^ μm^2^	88 ± 19	93 ± 29	75 ± 22	0.14	NA	NA	NA
LA, ×10^3^ μm^2^	57 ± 12	63 ± 18	52 ± 13	0.14	NA	NA	NA
SA, ×10^3^ μm^2^	31 ± 9	30 ± 12	23 ± 10	0.16	NA	NA	NA
L/C ratio, %	65.3 ± 6.0	68.3 ± 6.9	71.0 ± 7.2	0.07	NA	NA	NA
**Haller’s layer**							
CA, ×10^3^ μm^2^	258 ± 49	266 ± 98	195 ± 46	**0.02**	0.91	**0.005**	0.12
LA, ×10^3^ μm^2^	169 ± 38	175 ± 64	123 ± 31	**0.009**	0.86	**0.004**	0.053
SA, ×10^3^ μm^2^	89 ± 20	92 ± 35	72 ± 18	0.09	NA	NA	NA
L/C ratio, %	65.4 ± 6.3	65.4 ± 3.7	62.9 ± 3.9	0.49	NA	NA	NA
**CCT, μm**	264 ± 33	265 ± 77	203 ± 40	**0.003**	0.80	**<0.001**	**0.04**

CA, choroidal area; LA, luminal area; SA, stromal area; L/C ratio, luminal/choroidal ratio, CCT, central choroidal thickness; NA, not applicable. Statistically significant values are highlighted in bold.

**Table 4:** Postoperative BCVA and retinal structural changes in idiopathic macular holes (IMHs).

	Pre	Post 1M	Post 2M	Friedman Test (*p* Value)	Bonferroni Correction (*p* Value): Pre–Post 1M	Bonferroni Correction (*p* Value): Pre–Post 2M	Bonferroni Correction (*p* Value): Post 1M–Post 2M
**BCVA**							
IMH eyes with smooth borders	0.71 ± 0.28	0.46 ± 0.22	0.35 ± 0.25	**<0.001**	**0.002**	**<0.001**	0.052
IMH eyes with bumpy borders	0.86 ± 0.23	0.73 ± 0.21	0.62 ± 0.18	**0.01**	0.25	**0.04**	0.22
Mann–Whitney U test (*p* value)	0.17	**0.006**	**0.006**				
**ELM defect length, μm**							
IMH eyes with smooth borders	430 ± 198	77 ± 180	27 ± 125	**<0.001**	**<0.001**	**<0.001**	0.06
IMH eyes with bumpy borders	633 ± 179	333 ± 326	325 ± 308	0.24	NA	NA	NA
Mann–Whitney U test (*p* value)	**0.005**	**0.006**	**0.001**				
**EZ defect length, μm**							
IMH eyes with smooth borders	471 ± 162	235 ± 159	146 ± 122	**<0.001**	**<0.001**	**<0.001**	**0.003**
IMH eyes with bumpy borders	844 ± 286	651 ± 360	554 ± 381	**<0.001**	**0.03**	**0.02**	**0.02**
Mann–Whitney U test (*p* value)	**0.001**	**<0.001**	**0.001**				

BCVA, best-corrected visual acuity; IMH, idiopathic macular hole; M, month; ELM, external limiting membrane; EZ, ellipsoid zone; NA, not applicable. Statistically significant values are highlighted in bold.

**Table 5:** Postoperative changes in total choroid area in idiopathic macular holes (IMHs).

	Pre	Post 1M	Post 2M	Friedman Test (*p* Value)	Bonferroni Correction (*p* Value): Pre–Post 1M	Bonferroni Correction (*p* Value): Pre–Post 2M	Bonferroni Correction (*p* Value): Post 1M–Post 2M
**Total choroid**							
CA, ×10^3^ μm^2^							
IMH eyes with smooth borders	396 ± 117	410 ± 119	401 ± 126	0.74	NA	NA	NA
IMH eyes with bumpy borders	301 ± 60	294 ± 65	278 ± 68	0.14	NA	NA	NA
Mann–Whitney U test (*p* value)	**0.02**	**0.004**	**0.009**				
LA, ×10^3^ μm^2^							
IMH eyes with smooth borders	260 ± 77	273 ± 78	271 ± 83	0.96	NA	NA	NA
IMH eyes with bumpy borders	190 ± 38	193 ± 40	183 ± 43	0.34	NA	NA	NA
Mann–Whitney U test (*p* value)	**0.001**	**0.004**	**0.003**				
SA, ×10^3^ μm^2^							
IMH eyes with smooth borders	137 ± 41	137 ± 44	130 ± 45	0.26	NA	NA	NA
IMH eyes with bumpy borders	112 ± 22	100 ± 26	95 ± 25	0.29	NA	NA	NA
Mann–Whitney U test (*p* value)	0.06	**0.01**	**0.03**				
L/C ratio, %							
IMH eyes with smooth borders	65.3 ± 3.0	66.6 ± 2.3	67.6 ± 2.5	**0.01**	0.21	**0.002**	0.31
IMH eyes with bumpy borders	63.0 ± 1.4	66.1 ± 2.4	66.1 ± 1.8	**0.004**	**0.04**	**0.03**	0.99
Mann–Whitney U test (*p* value)	**0.005**	0.48	0.12				
**CCT, μm**							
IMH eyes with smooth borders	265 ± 77	276 ± 80	270 ± 85	0.76	NA	NA	NA
IMH eyes with bumpy borders	203 ± 40	196 ± 46	185 ± 47	**0.045**	0.64	**0.049**	0.46
Mann–Whitney U test (*p* value)	**0.02**	**0.004**	**0.006**				

M, month; CA, choroidal area; LA, luminal area; SA, stromal area; L/C ratio, luminal/choroidal ratio; CCT, central choroidal thickness; NA, not applicable. Statistically significant values are highlighted in bold.

**Table 6:** Postoperative changes in choriocapillaris in idiopathic macular holes (IMHs).

	Pre	Post 1M	Post 2M	Friedman Test (*p* Value)	Bonferroni Correction (*p* Value): Pre–Post 1M	Bonferroni Correction (*p* Value): Pre–Post 2M	Bonferroni Correction (*p* Value): Post 1M–Post 2M
**Choriocapillaris**							
CA, ×10^3^ μm^2^							
IMH eyes with smooth borders	37 ± 6	37 ± 6	40 ± 5	0.07	NA	NA	NA
IMH eyes with bumpy borders	32 ± 6	33 ± 8	36 ± 5	0.35	NA	NA	NA
Mann–Whitney U test (*p* value)	**0.03**	0.27	**0.02**				
LA, ×10^3^ μm^2^							
IMH eyes with smooth borders	23 ± 6	27 ± 6	30 ± 5	**<0.001**	**<0.001**	**<0.001**	**0.006**
IMH eyes with bumpy borders	15 ± 5	22 ± 6	25 ± 4	**<0.001**	**<0.001**	**<0.001**	**0.006**
Mann–Whitney U test (*p* value)	**0.003**	**0.04**	**0.002**				
SA, ×10^3^ μm^2^							
IMH eyes with smooth borders	15 ± 3	10 ± 2	10 ± 2	**<0.001**	**<0.001**	**<0.001**	0.82
IMH eyes with bumpy borders	17 ± 3	11 ± 3	11 ± 3	**0.004**	**0.006**	**0.006**	0.99
Mann–Whitney U test (*p* value)	0.09	0.67	0.43				
L/C ratio, %							
IMH eyes with smooth borders	60.1 ± 9.2	72.0 ± 6.3	75.9 ± 5.2	**<0.001**	**<0.001**	**<0.001**	0.06
IMH eyes with bumpy borders	46.9 ± 9.2	65.6 ± 8.0	70.4 ± 5.8	**<0.001**	**0.003**	**0.003**	0.07
Mann–Whitney U test (*p* value)	**0.002**	**0.02**	**0.02**				
IMH eyes with smooth borders	68.3 ± 6.9	67.7 ± 4.5	68.9 ± 4.6	0.68	NA	NA	NA
IMH eyes with bumpy borders	71.0 ± 7.2	71.1 ± 7.3	69.9 ± 6.4	0.23	NA	NA	NA
Mann–Whitney U test (*p* value)	0.26	0.25	0.87				

M, month; CA, choroidal area; LA, luminal area; SA, stromal area; L/C ratio, luminal/choroidal ratio; NA, not applicable. Statistically significant values are highlighted in bold.

**Table 7:** Postoperative changes in Sattler’s layer in idiopathic macular holes (IMHs).

	Pre	Post 1M	Post 2M	Friedman Test (*p* Value)	Bonferroni Correction (*p* Value): Pre–Post 1M	Bonferroni Correction (*p* Value): Pre–Post 2M	Bonferroni Correction (*p* Value): Post 1M–Post 2M
**Sattler’s layer**							
CA, ×10^3^ μm^2^							
IMH eyes with smooth borders	93 ± 29	101 ± 32	95 ± 31	0.57	NA	NA	NA
IMH eyes with bumpy borders	75 ± 22	93 ± 62	65 ± 20	0.06	NA	NA	NA
Mann–Whitney U test (*p* value)	0.07	0.07	**0.009**				
LA, ×10^3^ μm^2^							
IMH eyes with smooth borders	63 ± 18	68 ± 21	65 ± 21	0.74	NA	NA	NA
IMH eyes with bumpy borders	52 ± 13	64 ± 41	45 ± 11	0.06	NA	NA	NA
Mann–Whitney U test (*p* value)	0.09	**0.04**	**0.004**				
SA, ×10^3^ μm^2^							
IMH eyes with smooth borders	30 ± 12	33 ± 13	30 ± 12	0.40	NA	NA	NA
IMH eyes with bumpy borders	23 ± 10	29 ± 22	21 ± 10	0.53	NA	NA	NA
Mann–Whitney U test (*p* value)	0.16	0.11	**0.03**				
L/C ratio, %							
IMH eyes with smooth borders	68.3 ± 6.9	67.7 ± 4.5	68.9 ± 4.6	0.68	NA	NA	NA
IMH eyes with bumpy borders	71.0 ± 7.2	71.1 ± 7.3	69.9 ± 6.4	0.23	NA	NA	NA
Mann–Whitney U test (*p* value)	0.26	0.25	0.87				

M, month; CA, choroidal area; LA, luminal area; SA, stromal area; L/C ratio, luminal/choroidal ratio; CCT, central choroidal thickness; NA, not applicable. Statistically significant values are highlighted in bold.

**Table 8:** Postoperative changes in Haller’s layer in idiopathic macular holes (IMHs).

	Pre	Post 1M	Post 2M	Friedman Test (*p* Value)	Bonferroni Correction (*p* Value): Pre–Post 1M	Bonferroni Correction (*p* Value): Pre–Post 2M	Bonferroni Correction (*p* Value): Post 1M–Post 2M
**Haller’s layer**							
CA, ×10^3^ μm^2^							
IMH eyes with smooth borders	266 ± 98	278 ± 107	270 ± 103	0.84	NA	NA	NA
IMH eyes with bumpy borders	195 ± 46	189 ± 42	177 ± 54	0.44	NA	NA	NA
Mann–Whitney U test (*p* value)	0.053	**0.001**	**0.03**				
LA, ×10^3^ μm^2^							
IMH eyes with smooth borders	175 ± 64	182 ± 69	178 ± 67	0.96	NA	NA	NA
IMH eyes with bumpy borders	123 ± 31	121 ± 29	113 ± 36	0.70	NA	NA	NA
Mann–Whitney U test (*p* value)	**0.02**	**0.009**	**0.008**				
SA, ×10^3^ μm^2^							
IMH eyes with smooth borders	92 ± 35	97 ± 39	92 ± 37	0.88	NA	NA	NA
IMH eyes with bumpy borders	72 ± 18	68 ± 15	64 ± 19	0.18	NA	NA	NA
Mann–Whitney U test (*p* value)	0.14	**0.02**	**0.04**				
L/C ratio, %							
IMH eyes with smooth borders	65.4 ± 3.7	65.1 ± 2.8	65.8 ± 2.9	0.96	NA	NA	NA
IMH eyes with bumpy borders	62.9 ± 3.9	64.1 ± 3.1	63.6 ± 2.9	0.91	NA	NA	NA
Mann–Whitney U test (*p* value)	0.27	0.37	**0.04**				

M, month; CA, choroidal area; LA, luminal area; SA, stromal area; L/C ratio, luminal/choroidal ratio; NA, not applicable. Statistically significant values are highlighted in bold.

**Table 9:** Multiple regression analysis to identify preoperative factors predicting improvement in L/C ratio of choriocapillaris after macular hole surgery.

Variables	Correlation Coefficient	*p* Value
**Preoperative**		
Hole borders, smooth/bumpy	**0.73**	**0.01**
MH stage	−0.01	0.95
Minimum hole diameter	−0.62	0.07
Basal hole diameter	**1.27**	**0.02**
ELM defect length	0.01	0.63
EZ defect length, μm	0.02	0.09
Supra-RPE granular deposits	3.63	0.57

MH, macular hole; ELM, external limiting membrane; EZ, ellipsoid zone, RPE, retinal pigment epithelium. Statistically significant values are highlighted in bold.

**Table 10:** Clinical and structural differences between smooth and bumpy idiopathic macular hole borders.

Parameter	Smooth Border	Bumpy Border
Visual recovery	Early and favorable	Delayed and limited
Symptom duration	Short (28 ± 26 days)	Long (96 ± 73 days)
MH stage	Mainly stage 2–3	Mainly stage 3–4
Inner macular fluid	34.8%	100.0%
ELM/EZ defect length	Short	Long
Basal hole diameter	Small (666 ± 216 μm)	Large (978 ± 303 μm)
Supra-RPE granular deposits	34.8%	81.8%
Choriocapillaris L/C ratio	High (60.1 → 75.9%)	Low (46.9 → 70.4%)
Central choroidal thickness	Stable	Further decrease postoperative

MH, macular hole; ELM, external limiting membrane; EZ, Ellipsoid zone; RPE, retinal pigment epithelium; L/C ratio, luminal/choroidal ratio.

## Data Availability

The data that support the findings of this study are available on request from the corresponding authors, H.E. and Y.I. The data are not publicly available due to their containing information that could compromise the privacy of research participants.
